# Supplementary material for: HMGA1 orchestrates chromatin compartmentalization and sequesters genes into 3D networks coordinating senescence heterogeneity
Source: Nat Commun. 2024 Aug 12;15:6891. doi: 10.1038/s41467-024-51153-8 (PMC11319441; doi:10.1038/s41467-024-51153-8)
Supplement: Supplementary file 1 — Supplementary Information [file 41467_2024_51153_MOESM1_ESM.pdf]

Supplementary Information

Supplementary Notes

Network properties

The degree of a node is the number of connections between the node and the rest of the network.

Dense subgraphs

A subgraph corresponds to a subset of nodes and the edges with endpoints within the subset. A dense subgraph is characterised by a large number of edges relative to the number of nodes.

K-core decomposition

The *k*-core number established by Seidman (Seidman 1983). The *k*-core decomposition method classifies each node into layers, such that every node in the *k*-layer has a degree of at most *k*. The layers are calculated by sequentially removing nodes: first all disconnected nodes, then all nodes with only one connection (degree 1), until all nodes have been assigned to a 'shell', forming a nested structure of networks with the most densely connected subgraphs at its centre. This method has been successfully applied to characterising large, complex, real-world networks, such as protein-protein interactions, where proteins with large *k*-core values form complexes and are more evolutionarily conserved, or social networks, such as Twitter, where users with high *k*-core values are more influential (Kong *et al.* 2019).

Degeneracy core (k-max)

The maximal *k*-core number (*k*-max) is called the degeneracy of the graph and is a measure of sparsity, with low values indicating more disconnected graphs. The subgraph in which all nodes have *k*-max is subsequently called the degeneracy-core.

Supplementary Figure 1

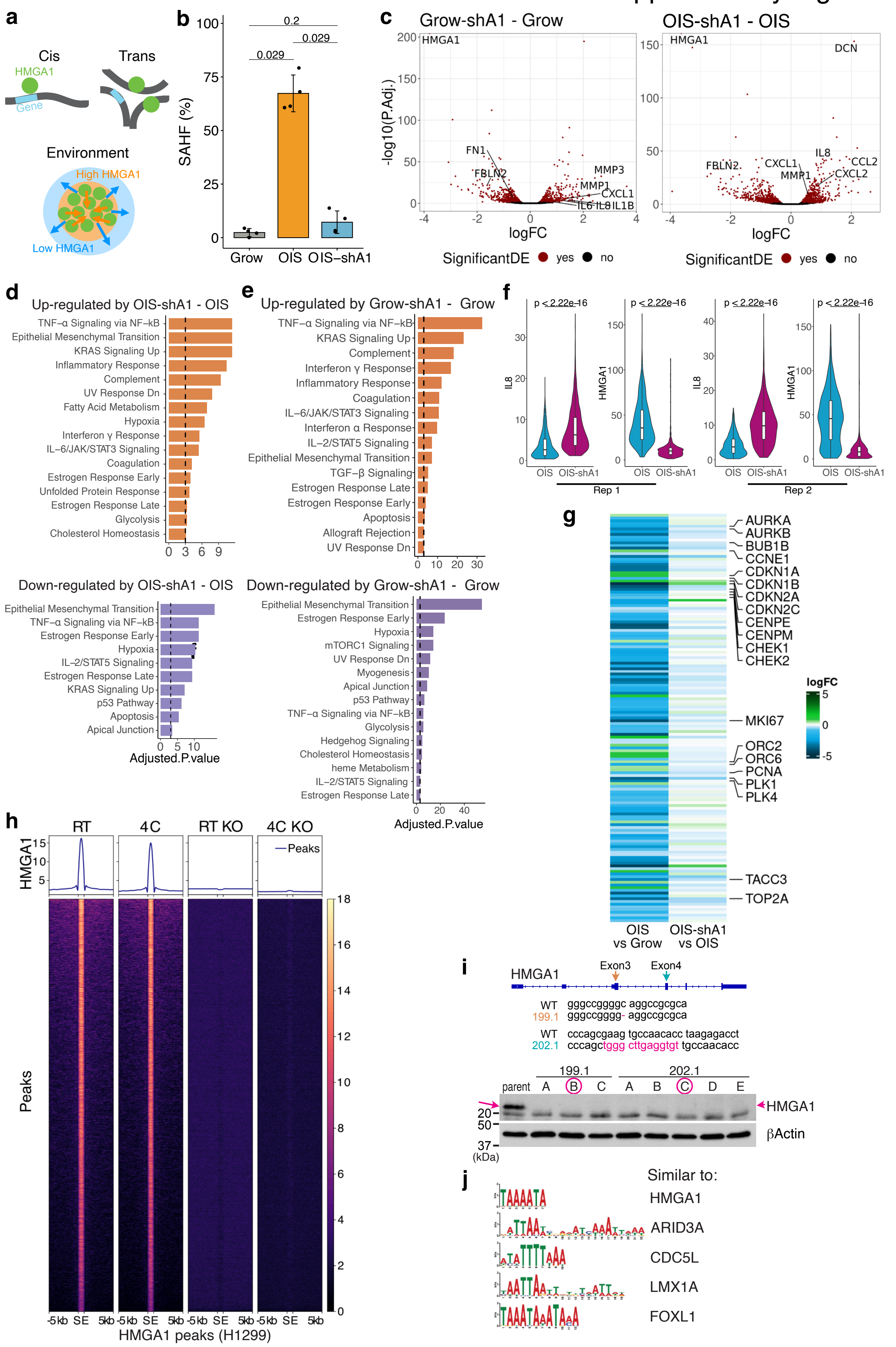

Supplementary Fig. 1: HMGA1-driven transcriptomic changes and genome-wide binding features. a, Representation of different gene regulatory modalities proposed for HMGA1: in *cis* by directly binding to the gene, in *trans* by binding near the gene or binding enhancers targeting the gene, and by modulating the chromatin environment. **b,** Percentage of cells with SAHF detected from DAPI nuclear staining in n=216 (Grow), n=183 (OIS), and n=189 (OIS-shA1) cells (4 biological replicates each). Error bars, mean ± s.d. P-values derived from two-sided Wilcoxon testing. **c,** Differential expression (DE) analysis in Grow and OIS with shA1 (n=8 replicates per condition): log-fold changes (logFC) against adjusted p-values (-log10(p-value)), highlighting significantly DE genes. P-values were derived from edgeR differential expression testing with multiple testing Benjamini-Hochberg correction. **d-e,** Gene set enrichment analysis against the MSigDB Hallmarks gene sets of genes up- and down-regulated in **d:** OIS-shA1 compared to OIS and **e:** Grow-shA1 compared to Grow. P-values were determined using Fischer's exact test and Benjamini-Hochberg multiple testing correction. **f,** Quantification of the immunofluorescence signal of IL-8 and HMGA1 averaged per cell (nucleus) in two replicates of OIS and OIS-shA1 (n=567 and 1,113 OIS cells and n=734 and 1,322 OIS-shA1 cells in each replicate). P-values derived from two-sided Wilcoxon testing. Box plot centre line represents the median, the bounds correspond to the 0.25 and 0.75 quantiles, the whiskers represent the 0.1 and 0.9 quantiles. **g,** Gene expression log-fold changes (logFC) in OIS compared to Grow and OIS-shA1 compared to OIS of the 'E2F targets' gene set from MSigDB. **h,** HMGA1 binding of the regions (10kb) centred around HMGA1 peaks in H1299 cells: with and without HMGA1 KO, under the room temperature (RT) or 4 degrees Celsius temperature (4C) fixation conditions (see Methods). **i,** HMGA1 protein levels in the H1299 cells with and without HMGA1 KO (n=8 KO clones, circled in red are the two clones we chose for the ChIP-seq experiments). Top, genotyping of corresponding clones. **j,** Motif discovery analysis of the top 40,000 HMGA1 peaks (highest signal) alongside proteins with a similar motif (right-side).

Supplementary Figure 2

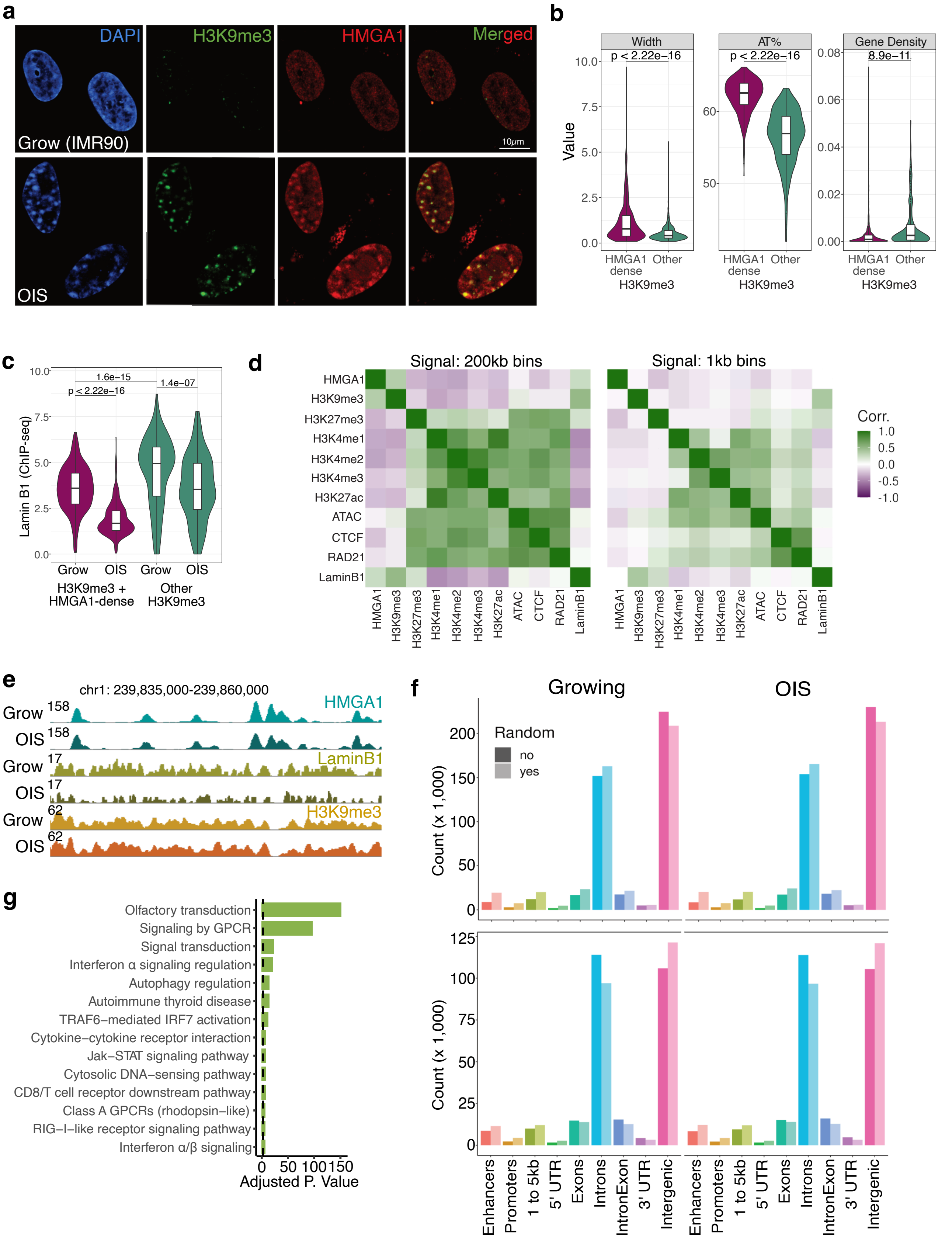

Supplementary Fig. 2: Gene promoters and enhancers bound by HMGA1. a, Immunofluorescence imaging of Grow and OIS cells (n=2 each): DAPI nuclear staining, H3K9me3 (green) and HMGA1 (red). **b,** Properties of H3K9me3 peaks with (n=546) and without overlap (n=217) with HMGA1-dense regions: width, average AT% and gene density. **c,** The distribution of the normalised ChIP-seq signal of Lamin B1 in the Grow and OIS conditions summarised over the H3K9me3 peaks with (n=546) and without overlap (n=217) with HMGA1-dense regions. **b-c**, P-values derived from two-sided Wilcoxon testing. Box plot centre line represents the median, the bounds correspond to the 0.25 and 0.75 quantiles, the whiskers represent the 0.1 and 0.9 quantiles. **d,** Correlation between normalised ChIP-seq (or ATAC-seq) signal summarised over 200kb bins (low-resolution, top) and 1kb bins (high-resolution, bottom) of HMGA1 and selected proteins and histone marks. **e,** ChIP-seq normalised signal tracks (IGV) of HMGA1, Lamin B1 and H3K9me3 in the Grow and OIS conditions of a randomly chosen genomic location within a H3K9me3 peak with HMGA1 and LaminB1 binding**. f,** Annotation of HMGA1 peaks (top - all peaks, bottom - excluding peaks overlapping H3K9me3) in the Grow (left) and OIS (right) conditions, against enhancers (defined previously) and genic elements. **g,** Gene set enrichment analysis of the genes whose promoters are bound by HMGA1 in Grow using the BioPlanet gene sets (EnrichR). P-values were determined using Fischer's exact test and Benjamini-Hochberg multiple testing correction.

Supplementary Figure 3

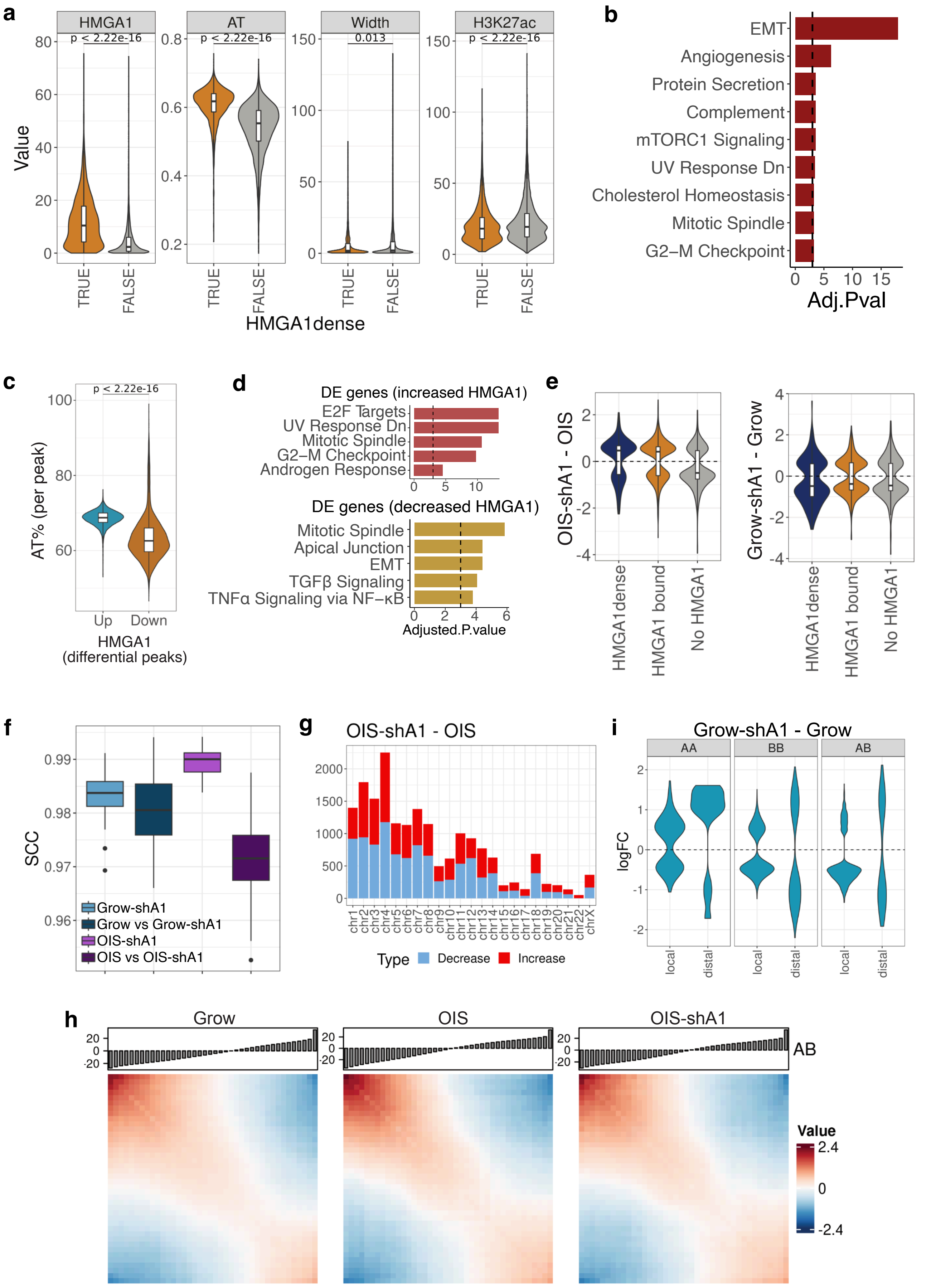

Supplementary Fig. 3: HMGA1-dependent differential interactions. a, Sequence and epigenetic properties (normalised ChIP-seq signal) of enhancers with (n=3,429) and without overlap (n=19,305) with HMGA1 dense regions; the scale of the enhancers width is in Mb. **b,** Gene set enrichment results of the genes expressed in IMR90 cells with promoters proximal (within 50kb) of the HMGA1-dense enhancers. **c,** The average AT% of: the peaks with differential HMGA1 binding (n=16,420 increased - Up, n=4,060 decreased - Down). **d,** Gene set enrichment analysis results (top 5 results) using the MSigDB Hallmarks gene sets of the genes DE in OIS compared to Grow which have increased (top) and decreased (bottom) HMGA1 binding. P-values were determined using Fischer's exact test and Benjamini-Hochberg multiple testing correction. **e,** Distribution of expression changes (logFC) of genes within HMGA1-dense regions, with any HMGA1 binding, and lacking HMGA1 binding in the OIS-shA1 compared to OIS (left, n=218, 518, and 445) and Grow-shA1 compared to Grow (right, n=213, 380, and 286 genes, respectively) comparisons. **f,** Stratum-adjusted correlation coefficient (SCC) between the Hi-C libraries of the Grow-shA1 biological replicates (n=2), the OIS-shA1 replicates (n=2) and between the Grow and Grow-shA1 samples and OIS and OIS-shA1 samples; 1 indicates maximum agreement. **g,** The number of interaction changes per chromosome in OIS-shA1 compared to OIS, separated by increases (red) and decreases (blue). **h,** Saddle-plots of the normalised and distance-corrected matrices of contact frequency in the Grow, OIS and OIS-shA1 conditions, stratified by A/B compartment scores. **i,** Distribution of interaction log-fold changes in Grow-shA1 - Grow classified by the A/B compartment status and the distance between the interacting regions: local (less than 2Mb) and distal (greater than 2Mb) between the regions. P-values derived from two-sided Wilcoxon testing. Box plot centre line represents the median, the bounds correspond to the 0.25 and 0.75 quantiles, the whiskers represent the 0.1 and 0.9 quantiles.

Supplementary Figure 4

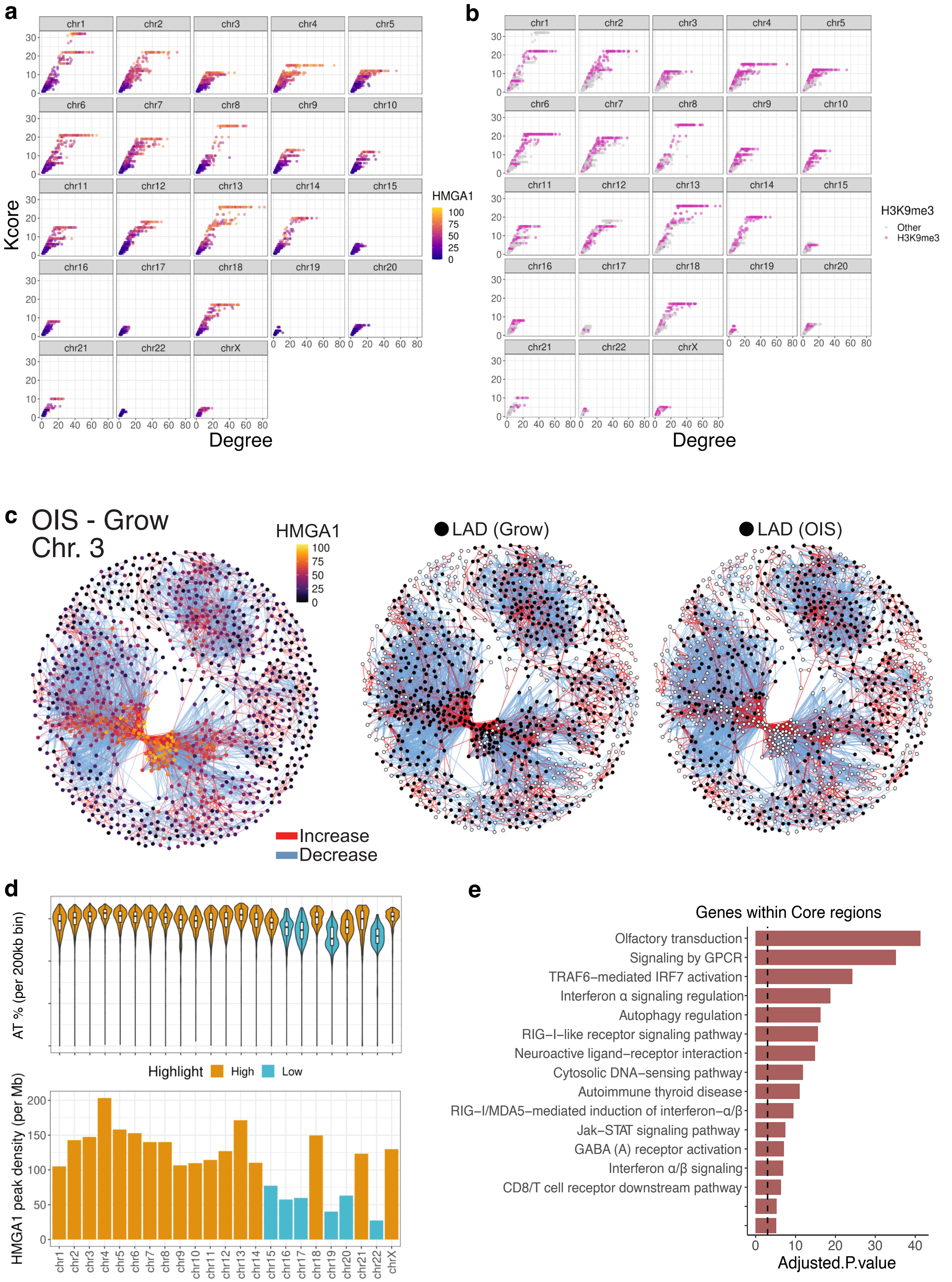

Supplementary Fig. 4: The *k*-core layering of the networks of increased interactions in OIS compared to Grow. a-b, The degree (number of interactions) against the *k*-core of each node in the chromosomal networks of increased interactions in OIS (compared to Grow), highlighting **a,** HMGA1 binding (normalised ChIP-seq signal averaged over the 200kb bind corresponding to the nodes) and **b,** overlap status of the nodes (bins) with H3K9me3 peaks. **c,** Differential interactions network of chromosome 3 comparing the OIS and Grow conditions, highlighting the overlap between the bins (nodes) with: left - HMGA1 peaks (OIS), middle - Lamin B1 associated domains (LAD) in the Grow condition, and right - LAD in the OIS condition (black nodes - overlapping LAD, white nodes - no overlap with LAD). **d,** The distribution of the AT content (top, 0 - no AT, 1 - no GC) and bottom: the HMGA1 peak density (bottom, scaled per Mb) of each chromosome, highlighting chromosomes with low AT content or low HMGA1 density. P-values derived from two-sided Wilcoxon testing. Box plot centre line represents the median, the bounds correspond to the 0.25 and 0.75 quantiles, the whiskers represent the 0.1 and 0.9 quantiles. **e,** Gene enrichment results (BioPlanet gene sets) of all the genes within 'Core' regions. P-values were determined using Fischer's exact test and Benjamini-Hochberg multiple testing correction.

Supplementary Figure 5

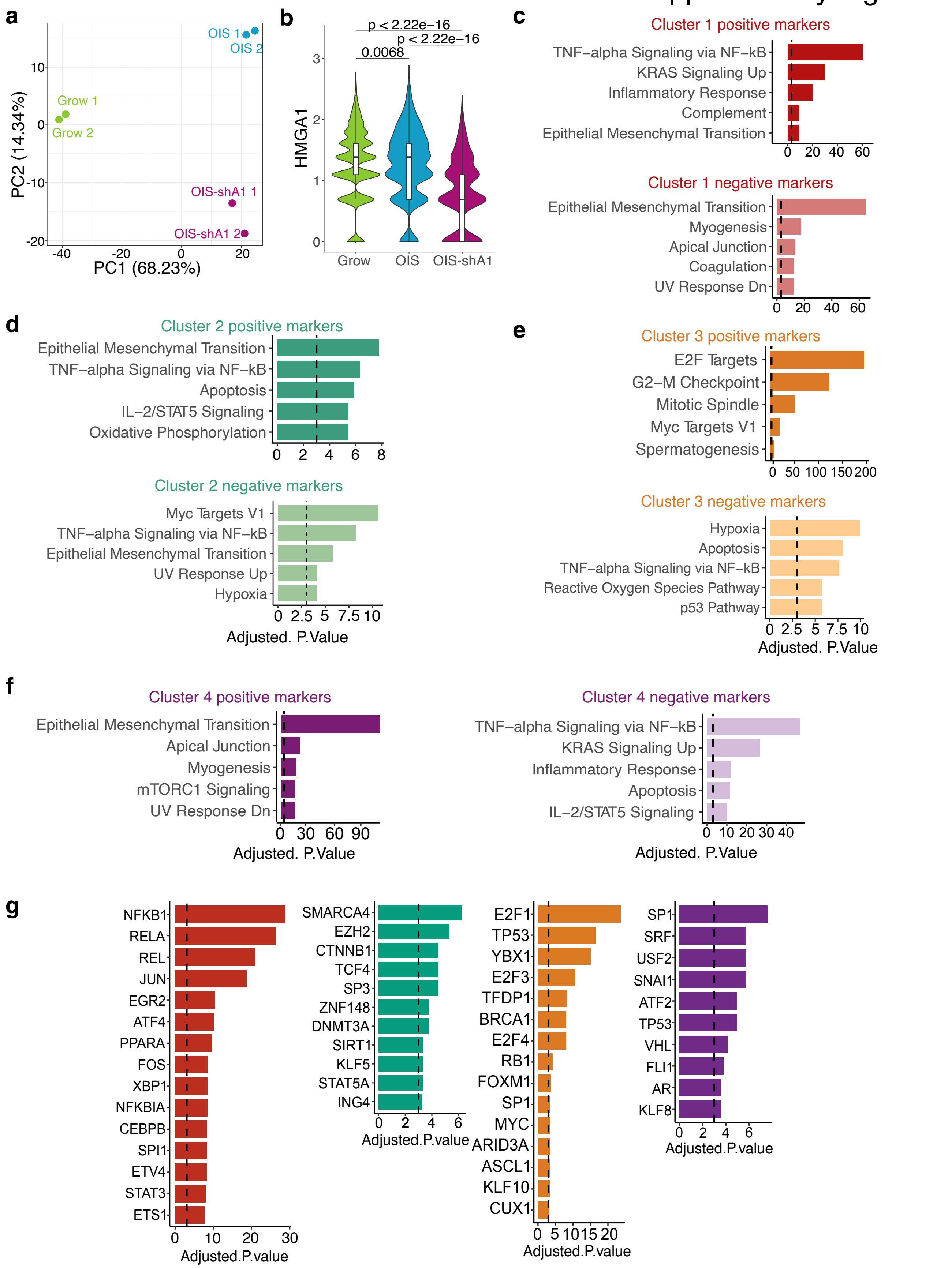

Supplementary Fig. 5: Characterisation of HMGA1-dependent transcriptional changes at single-cell level. a, Principal component analysis of the pseudo-bulk scRNA-seq samples of the Grow, OIS, and OIS-shA1 conditions with two replicates each. **b,** Distribution of HMGA1 expression in the n=6,165 Grow, n=2,828 OIS, and n=2,073 OIS-shA1 cells. P-values derived from two-sided Wilcoxon testing. Box plot centre line represents the median, the bounds correspond to the 0.25 and 0.75 quantiles, the whiskers represent the 0.1 and 0.9 quantiles. **c-f,** The top MSigDB Hallmarks enriched from the positive and negative markers of the cell neighbourhoods in **c,** cluster 1, **d,** cluster 2, **e,** cluster 3, and **f,** cluster 4. **g,** Transcription factors (TFs) enriched based on the TRRUST TF-target database as potential regulators of the positive marker genes in clusters 1-4. P-values were determined using Fischer's exact test and Benjamini-Hochberg multiple testing correction.

Supplementary Figure 6

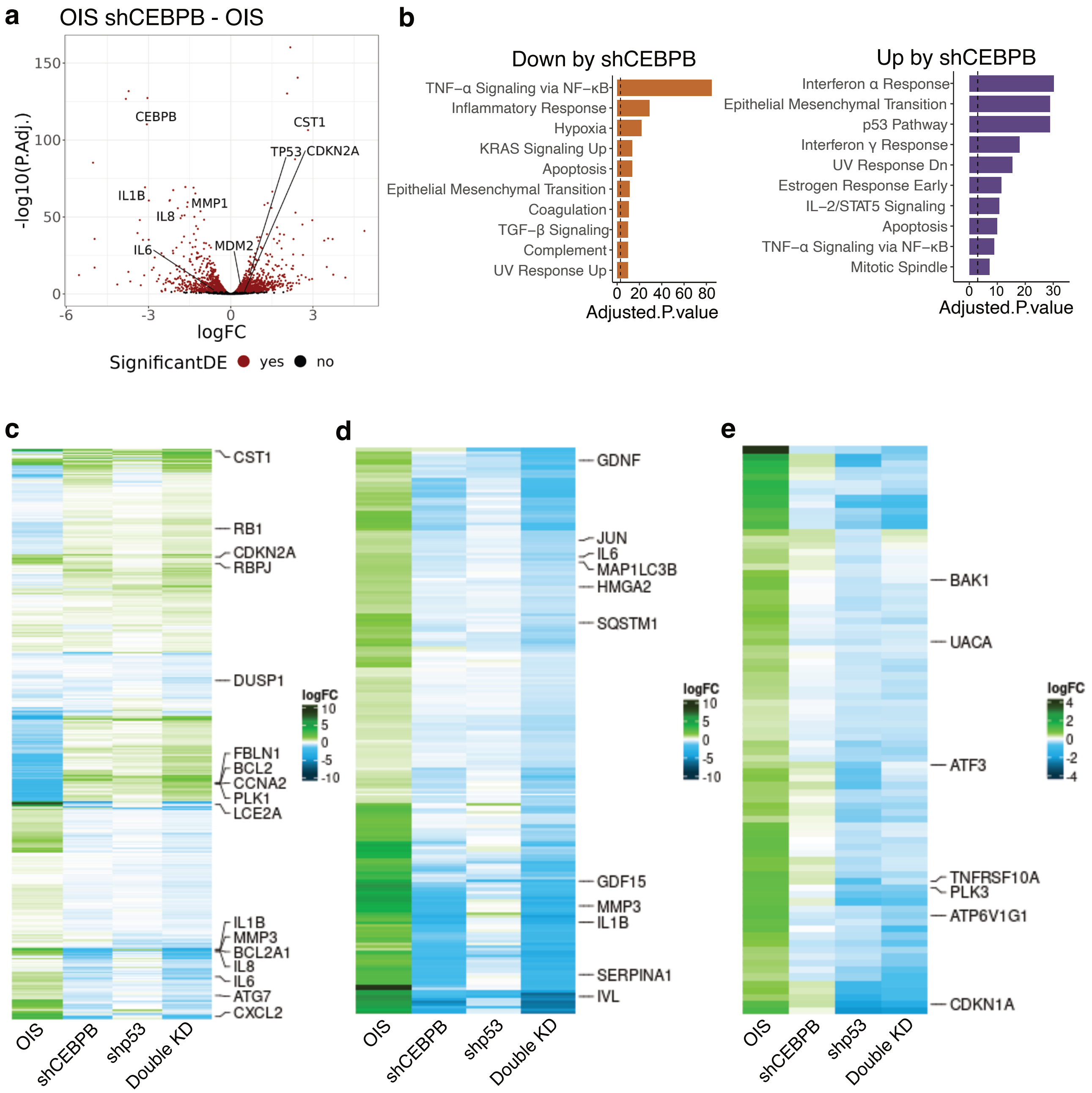

Supplementary Fig. 6: The combined regulatory effect of CEBPB and p53 in OIS. a, Differential expression in OIS shCEBPB compared to OIS, highlighting the DE genes. **b,** Gene enrichment results (MSigDb Hallmarks) of the genes down-regulated (left) and up-regulated (right) by shCEBPB in OIS. P-values were calculated using the statistical testing for differential expression implemented in the R package edgeR and corrected for multiple testing with the Benjamini-Hochberg adjustment. **c,** Expression log-fold changes in OIS (compared to Grow), OIS shCEBPB (compared to OIS), OIS shp53 (compared to OIS), and OIS with double knock-down of CEBPB and p53 (compared to OIS) of the genes DE in at least one comparison. **d-e,** Genes (log-fold changes) whose alteration is either more pronounced or attenuated by the double knock-down compared to the individual effect of **d,** shCEBPB and **e,** shp53. P-values were determined using Fischer's exact test and Benjamini-Hochberg multiple testing correction.

Supplementary References:

1. Seidman, S. B. Network structure and minimum degree. *Social Networks* 5, 269–287 (1983).

2. Kong, Y *et al.* k-core: Theories and applications. *Physics Reports* 832, 1–32 (2019).
