# Supplementary material for: HMGA1 orchestrates chromatin compartmentalization and sequesters genes into 3D networks coordinating senescence heterogeneity
Source: Nat Commun. 2024 Aug 12;15:6891. doi: 10.1038/s41467-024-51153-8 (PMC11319441; doi:10.1038/s41467-024-51153-8)
Supplement: Supplementary file 3 — Reporting Summary [file 41467_2024_51153_MOESM3_ESM.pdf]

nature portfolio

Corresponding author(s): Masashi Narita

Last updated by author(s): 22/07/2024

Reporting Summary

Nature Portfolio wishes to improve the reproducibility of the work that we publish. This form provides structure for consistency and transparency in reporting. For further information on Nature Portfolio policies, see our Editorial Policies and the Editorial Policy Checklist.

Statistics

For all statistical analyses, confirm that the following items are present in the figure legend, table legend, main text, or Methods section.

n/a	Confirmed	
☐	☒	The exact sample size (*n*) for each experimental group/condition, given as a discrete number and unit of measurement
☐	☒	A statement on whether measurements were taken from distinct samples or whether the same sample was measured repeatedly
☐	☒	The statistical test(s) used AND whether they are one- or two-sided<br>*Only common tests should be described solely by name; describe more complex techniques in the Methods section.*
☐	☒	A description of all covariates tested
☐	☒	A description of any assumptions or corrections, such as tests of normality and adjustment for multiple comparisons
☐	☒	A full description of the statistical parameters including central tendency (e.g. means) or other basic estimates (e.g. regression coefficient) AND variation (e.g. standard deviation) or associated estimates of uncertainty (e.g. confidence intervals)
☐	☒	For null hypothesis testing, the test statistic (e.g. *F*, *t*, *r*) with confidence intervals, effect sizes, degrees of freedom and *P* value noted<br>*Give P values as exact values whenever suitable.*
☒	☐	For Bayesian analysis, information on the choice of priors and Markov chain Monte Carlo settings
☒	☐	For hierarchical and complex designs, identification of the appropriate level for tests and full reporting of outcomes
☐	☒	Estimates of effect sizes (e.g. Cohen's *d*, Pearson's *r*), indicating how they were calculated

Our web collection on statistics for biologists contains articles on many of the points above.

Software and code

Policy information about availability of computer code

Data collection	No software was used.
Data analysis	No specialised code was used for this analysis, beyond basic R functionality (all packages used mentioned in the Methods section) for calculations and visualisation. ChIP-seq analysis: Reads were aligned using Bowtie2 (v.2.2.4) against the hg19 genome build. No trimming was required as determined with FastQC (v.0.12, Simon Andrews). Duplicates were marked with samtools (v.1.9) and blacklisted regions (hg19) were removed. Samples were checked for quality control metrics and extension sizes were calculated for each sample with the R Bioconductor package ChIPQC (v.1.32.2). Peaks were called with macs3 (v.3.0.0) initially with default parameters and then with the extension sizes determined. Peaks present in at least two of the replicates of a condition were combined into a consensus set for each condition. THOR (v.1.0.2) was used to normalise and to perform differential binding pairwise between conditions. The THOR-normalised signal was used for visualisation with the IGV genome browser. HMGA1-dense regions were calculated by counting the number of peaks in rolling windows consisting of 100kb bins with a step of 5kb and then computing the inflection point of these values beyond which a region was classified as ‘dense’. ChIP-seq heatmaps were plotted using deepTools (v.3.1.0). Peak annotation was performed with the R Bioconductor package annotatR (v.1.22.0). Bulk RNA-seq analysis: Libraries were aligned using STAR (v.2.7.10) against the hg19 genome and transcriptome (GENCODE19) reference. We used n=8 replicates per condition. Reads were counted over genes using the GENCODE19 annotation and the featureCounts functionality from the subread package (v.1.6.2). Differential expression analysis was performed pairwise using the R Bioconductor package edgeR (v.3.40.2). Gene set enrichment was performed using the R interface of EnrichR, the enrichR package (v.3.2). scRNA-seq analysis: Single-cell RNA-seq libraries were processed using the CellRanger pipeline (10x Genomics) against the hg19 reference transcriptome. Read counts were further analysed using the R Bioconductor package Seurat (v.4.1.0). Hashtag demultiplexing was performed with HTODemux from Seurat and doublet identification was performed with DoubletFinder using the hashtag read counts. After selecting singlets, low quality cells were filtered out (mitochondrial content above 5%). We used the edgeR Bioconductor package (v.3.40.2) for pseudobulk analysis. The Milo R Bioconductor package (v.1.4.0) was used for differential analysis testing on cell neighbourhoods. Gene set

scores were calculated using UCell (v.2.2.0). Hi-C analysis: Libraries were aligned using HiC-Pro (v.3.1.0) against the hg19 genome. Experimental artefacts and duplicates were removed prior to counting reads in matrices corresponding to different resolutions. Agreement between replicates was testing with HiCRep (v.1.12.2), as well as by principal component analysis of the normalised and filtered count matrices at several resolutions. A/B compartment score was determined as before, by performing PCA on distance-corrected ICE-normalized Hi-C matrices at 200kb resolution using the R Bioconductor package diffHic (v.1.28.0). Differential interaction analysis was performed using the same package (diffHic). Network analysis of the differential interactions was performed using the igraph R package (v. 1.3.5, CRAN Csardi & Nepusz, 2006). The ggraph R package (v.2.2.0, Pedersen 2022 CRAN) was used for plotting the chromosomal networks. ICE-normalised and distance-corrected matrices were used for visualisation using HiCvizR (v.1.0), as described before. Image analysis: Immunofluorescence signal quantification was performed with the StarDist (v.0.8.3 nuclei segmentation) and the scikit-image (v.0.19.3 image processing) Python libraries. HMGA1 average signal was calculated for each labelled cell nucleus and the IL-8 average signal was calculated over an area around the nuclear boundary.

For manuscripts utilizing custom algorithms or software that are central to the research but not yet described in published literature, software must be made available to editors and reviewers. We strongly encourage code deposition in a community repository (e.g. GitHub). See the Nature Portfolio guidelines for submitting code & software for further information.

Data

Policy information about availability of data

All manuscripts must include a data availability statement. This statement should provide the following information, where applicable:

- Accession codes, unique identifiers, or web links for publicly available datasets
- A description of any restrictions on data availability
- For clinical datasets or third party data, please ensure that the statement adheres to our policy

Source data are provided as a Source Data file. All sequencing data generated during this study are available from the Gene Expression Omnibus (GEO) under the accession ID GSE245808 [https://www.ncbi.nlm.nih.gov/geo/query/acc.cgi?acc=GSE245808]. It contains bulk RNA-seq, scRNA-seq, Hi-C and ChIP-seq data. We also used the following datasets which we previously published and deposited on GEO: Hi-C IMR90, CTCF and RAD21 ChIP-seq - GSE1350938 [https://www.ncbi.nlm.nih.gov/geo/query/acc.cgi?acc=GSE135093], Lamin B1 ChIP-seq – GSE4934122 [https://www.ncbi.nlm.nih.gov/geo/query/acc.cgi?acc= GSE49341], histone marks and ATAC-seq - GSE10359016 [https://www.ncbi.nlm.nih.gov/geo/query/acc.cgi?acc=GSE103590] and GSE3844271 [https://www.ncbi.nlm.nih.gov/geo/query/acc.cgi?acc=GSE38442], p53 ChIP-seq - GSE5349136 [https://www.ncbi.nlm.nih.gov/geo/query/acc.cgi?acc=GSE53491], and C/EBP� ChIP-seq - GSE18035817 [https://www.ncbi.nlm.nih.gov/geo/query/acc.cgi?acc= GSE180358]. Other public datasets (single cell RNA-seq) we used are: GSE13190740 [https://www.ncbi.nlm.nih.gov/geo/query/acc.cgi?acc=GSE131907] (lung adenocarcinoma) and GSE18359039 [https://www.ncbi.nlm.nih.gov/geo/query/acc.cgi?acc=GSE183590] (H1299 cell line).

Research involving human participants, their data, or biological material

Policy information about studies with human participants or human data. See also policy information about sex, gender (identity/presentation), and sexual orientation and race, ethnicity and racism.

Reporting on sex and gender	*Use the terms sex (biological attribute) and gender (shaped by social and cultural circumstances) carefully in order to avoid confusing both terms. Indicate if findings apply to only one sex or gender; describe whether sex and gender were considered in study design; whether sex and/or gender was determined based on self-reporting or assigned and methods used. Provide in the source data disaggregated sex and gender data, where this information has been collected, and if consent has been obtained for sharing of individual-level data; provide overall numbers in this Reporting Summary. Please state if this information has not been collected. Report sex- and gender-based analyses where performed, justify reasons for lack of sex- and gender-based analysis.*
Reporting on race, ethnicity, or other socially relevant groupings	*Please specify the socially constructed or socially relevant categorization variable(s) used in your manuscript and explain why they were used. Please note that such variables should not be used as proxies for other socially constructed/relevant variables (for example, race or ethnicity should not be used as a proxy for socioeconomic status). Provide clear definitions of the relevant terms used, how they were provided (by the participants/respondents, the researchers, or third parties), and the method(s) used to classify people into the different categories (e.g. self-report, census or administrative data, social media data, etc.) Please provide details about how you controlled for confounding variables in your analyses.*
Population characteristics	*Describe the covariate-relevant population characteristics of the human research participants (e.g. age, genotypic information, past and current diagnosis and treatment categories). If you filled out the behavioural & social sciences study design questions and have nothing to add here, write "See above."*
Recruitment	*Describe how participants were recruited. Outline any potential self-selection bias or other biases that may be present and how these are likely to impact results.*
Ethics oversight	*Identify the organization(s) that approved the study protocol.*

Note that full information on the approval of the study protocol must also be provided in the manuscript.

Field-specific reporting

Please select the one below that is the best fit for your research. If you are not sure, read the appropriate sections before making your selection.

☒ Life sciences ☐ Behavioural & social sciences ☐ Ecological, evolutionary & environmental sciences

For a reference copy of the document with all sections, see nature.com/documents/nr-reporting-summary-flat.pdf

Life sciences study design

All studies must disclose on these points even when the disclosure is negative.

Sample size	Sample sizes for ChIP-seq and Hi-C experiments were determined by comparison with previous studies and by taking into consideration the fact that we are studying one condition (RAS-induced senescence) against control (growing, proliferating cells). The dynamic range of DNA binding of the protein studied, HMGA1, appeared saturated based on the number of common peaks between replicates and on the Pearson correlation coefficients between normalised signal tracks. The Hi-C replicates were checked and found to agree well (as determined with two independent methods) and cis/trans interactions ratios similar to other studies.
Data exclusions	No data was excluded. All attempts at replication were successful.
Replication	We used HiCRep in order to check the agreement between Hi-C replicates. For ChIP-seq experiments, we checked agreement between replicates in terms of number of common peaks detected and overall Pearson correlation, calculated genome-wide using 1kb and 200kb bins.
Randomization	All the comparisons we performed were pairwise between control and senescent cells or between control and treatment (HMGA1 KO/ HMGA1 knockdown). We prioritized handling samples in ways which ensure no accidental mislabeling or switching can occur and batch effects are minimized. The readout from all the experiments was sequencing. Plate layout for library preparation and barcode assignment for sequencing was randomized using base R functions to score how well distributed are the conditions and replicates and determine optimal layouts.
Blinding	Blinding is not applicable in our case because no grouping was present besides the pairwise conditions studied, which have to be prepared in different ways and cannot be mislabeled.

Reporting for specific materials, systems and methods

We require information from authors about some types of materials, experimental systems and methods used in many studies. Here, indicate whether each material, system or method listed is relevant to your study. If you are not sure if a list item applies to your research, read the appropriate section before selecting a response.

Materials & experimental systems

n/a	Involved in the study	
☐	☒	Antibodies
☐	☒	Eukaryotic cell lines
☒	☐	Palaeontology and archaeology
☒	☐	Animals and other organisms
☒	☐	Clinical data
☒	☐	Dual use research of concern
☒	☐	Plants

Methods

n/a	Involved in the study	
☐	☒	ChIP-seq
☒	☐	Flow cytometry
☒	☐	MRI-based neuroimaging

Antibodies

Antibodies used	The following primary antibodies were used for immunoblotting: anti-β-actin (mouse monoclonal, Sigma, A5441 (AC-15), 1:2,500); anti-HRAS (mouse monoclonal, Santa Cruz, sc-29 (F235), 1:1,000); anti-HMGA1 (rabbit polyclonal, Active motif, Cat #39615, 1:2,000); anti-IL6 (mouse monoclonal, R&D Systems, MAB2061, 1:500); anti-IL8 (mouse monoclonal, R&D Systems, MAB208, 1:500); anti-Cyclin A (mouse monoclonal, Sigma, C4710 (CY-A1), 1:1000); anti-C/EBPb (rabbit polyclonal, Santa Cruz, sc-150 (C-19), 1:500); anti-p53 (mouse monoclonal, Santa Cruz, sc-126 (DO-1), 1:500); anti-CDKN1A p21 (mouse monoclonal, Santa Cruz, sc-6246 (F-5), 1:500); anti-CDKN2A p16 (mouse monoclonal, BD Biosciences 554079, clone G175-1239, 1:500).<br><br>The following primary antibodies were used for immunofluorescence: anti-H3K9me3 (mouse monoclonal, Hiroshi Kimura Laboratory, clone CMA318, 4µg/mL), anti-HMGA1 (rabbit polyclonal, Cold Spring Harbor Labs, CS2637, 1:1,000) and anti-IL8 (mouse monoclonal, R&D Systems, MAB208, 1:500).
Validation	anti-β-actin (mouse monoclonal, Sigma, A5441 (AC-15), 1:2,500); Manufacturer: This Antibody was verified by Knockdown to ensure that the antibody binds to the antigen stated. Used in: The novel anti-colitic effect of β-adrenergic receptors via modulation of PS1/ BACE-1/Aβ axis and NOTCH signaling in an ulcerative colitis model, Nasser et al., Front Pharmacol. 2022 Oct 25:13:1008085. doi: 10.3389/fphar.2022.1008085. eCollection 2022.

anti-HRAS (mouse monoclonal, Santa Cruz, sc-29 (F235), 1:1,000); Used in: Feedback activation of EGFR/wild-type RAS signaling axis limits KRAS G12D inhibitor efficacy in KRAS G12D-mutated colorectal cancer, Feng, J. et al. 2023. Oncogene. 42: 1620-1633.

anti-HMGA1 (rabbit polyclonal, Active motif, Cat #39615, 1:2,000); Used in: LINC00152 acts as a competing endogenous RNA of HMGA1 to promote the growth of gastric cancer cells, Chen et al., J Clin Lab Anal. 2022 Feb;36(2):e24192. doi: 10.1002/jcla.24192. Epub 2022 Jan 11. PMID: 35014092

anti-IL6 (mouse monoclonal, R&D Systems, MAB2061, 1:500); Used in: Head and neck squamous cancer cells enhance the differentiation of human mesenchymal stem cells to adipogenic and osteogenic lineages in vitro, Meyer et al., Oncol Lett. 2022 Oct 31;24(6):450. doi: 10.3892/ol.2022.13570. eCollection 2022 Dec. PMID: 36420071

anti-IL8 (mouse monoclonal, R&D Systems, MAB208, 1:500); Used in: Discovery of senolytics using machine learning, Smer-Barreto et al., Nat Commun. 2023 Jun 10;14(1):3445. doi: 10.1038/s41467-023-39120-1. PMID: 37301862

anti-Cyclin A (mouse monoclonal, Sigma, C4710 (CY-A1), 1:1000); Used in: Protein phosphatase 1 acts as a RIF1 effector to suppress DSB resection prior to Shieldin action, Isobe et al., Cell Rep. 2021 Jul 13; 36(2): 109383. PMID: 34260925

anti-C/EBPb (rabbit polyclonal, Santa Cruz, sc-150 (C-19), 1:500); Used in: Expression, hormonal regulation, and subcellular localization of CCAAT/enhancer-binding protein-beta in rat and human thyrocytes, Pomerance et al., Thyroid. 2005 Mar;15(3):197-204. doi: 10.1089/thy.2005.15.197. PMID: 15785238

anti-p53 (mouse monoclonal, Santa Cruz, sc-126 (DO-1), 1:500); Used in: The apelin□apelin receptor signaling pathway in fibroblasts is involved in tumor growth via p53 expression of cancer cells, Saiki et al., Int J Oncol. 2023 Dec;63(6):139. doi: 10.3892/ijo.2023.5587. Epub 2023 Nov 3. PMID: 37921070

anti-CDKN1A p21 (mouse monoclonal, Santa Cruz, sc-6246 (F-5), 1:500); Used in: FBXL12 degrades FANCD2 to regulate replication recovery and promote cancer cell survival under conditions of replication stress, Brunner et al., Mol Cell. 2023 Oct 19;83(20):3720-3739.e8. doi: 10.1016/j.molcel.2023.07.026. Epub 2023 Aug 16. PMID: 37591242

anti-H3K9me3 (mouse monoclonal, Hiroshi Kimura Laboratory, clone CMA318, 4µg/mL): Used in Kimura et al., The Organization of Histone H3 Modifications as Revealed by a Panel of Specific Monoclonal Antibodies. Cell Struct. Funct. 33, 61–73 (2008). PMID: 18227620

anti-HMGA1 (rabbit polyclonal, Cold Spring Harbor Labs, CS2637, 1:1,000): Used in Parry et al. NOTCH-mediated non-cell autonomous regulation of chromatin structure during senescence. Nat Commun. 2018 May 9;9(1):1840. doi: 10.1038/s41467-018-04283-9. PMID: 29743479

anti-CDKN2A p16 (mouse monoclonal, BD Biosciences 554079, clone G175-1239, 1:500):Used in Molkentine et al. p16 Represses DNA Damage Repair via a Novel Ubiquitin-Dependent Signaling Cascade. Cancer Res. 2022 Mar 1;82(5):916-928. doi: 10.1158/0008-5472.CAN-21-2101.

Eukaryotic cell lines

Policy information about cell lines and Sex and Gender in Research

Cell line source(s)	IMR90 human diploid fibroblasts (CCL-186) and H1299 human non-small cell lung cancer (NSCLC) cells (CRL-5803) were obtained from the American Type Culture Collection (ATCC).
Authentication	Cell identity was confirmed by STR (short tandem repeats) genotyping.
Mycoplasma contamination	Cells were regularly tested for mycoplasma contamination and always found to be negative.
Commonly misidentified lines (See ICLAC register)	No commonly misidentified cell lines were used.

Plants

Seed stocks	*Report on the source of all seed stocks or other plant material used. If applicable, state the seed stock centre and catalogue number. If plant specimens were collected from the field, describe the collection location, date and sampling procedures.*
Novel plant genotypes	*Describe the methods by which all novel plant genotypes were produced. This includes those generated by transgenic approaches, gene editing, chemical/radiation-based mutagenesis and hybridization. For transgenic lines, describe the transformation method, the number of independent lines analyzed and the generation upon which experiments were performed. For gene-edited lines, describe the editor used, the endogenous sequence targeted for editing, the targeting guide RNA sequence (if applicable) and how the editor was applied.*
Authentication	*Describe any authentication procedures for each seed stock used or novel genotype generated. Describe any experiments used to assess the effect of a mutation and, where applicable, how potential secondary effects (e.g. second site T-DNA insertions, mosiacism, off-target gene editing) were examined.*

ChIP-seq

Data deposition

☒ Confirm that both raw and final processed data have been deposited in a public database such as GEO.

☒ Confirm that you have deposited or provided access to graph files (e.g. BED files) for the called peaks.

Data access links<br>*May remain private before publication.*	All sequencing data generated during this study are available from the Gene Expression Omnibus (GEO) under the accession ID GSE245808.
Files in database submission	Grow_HMGA1_1.fq.gz<br>Grow_HMGA1_2.fq.gz<br>Grow_Input_1.fq.gz<br>Grow_Input_2.fq.gz<br>RIS_HMGA1_1.fq.gz<br>RIS_HMGA1_2.fq.gz<br>RIS_Input_1.fq.gz<br>RIS_Input_2.fq.gz<br>HMGA1_H1299_1.s_4.r_1.fq.gz HMGA1_H1299_1.s_5.r_1.fq.gz<br>HMGA1_H1299_2.s_4.r_1.fq.gz HMGA1_H1299_2.s_5.r_1.fq.gz<br>HMGA1_H1299_3.s_4.r_1.fq.gz HMGA1_H1299_3.s_5.r_1.fq.gz<br>Input_H1299_1.s_4.r_1.fq.gz Input_H1299_1.s_5.r_1.fq.gz<br>Input_H1299_2.s_4.r_1.fq.gz Input_H1299_2.s_5.r_1.fq.gz<br>Input_H1299_3.s_4.r_1.fq.gz Input_H1299_3.s_5.r_1.fq.gz<br>HMGA1_H1299_DoxshHMGA1_1.s_4.r_1.fq.gz HMGA1_H1299_DoxshHMGA1_1.s_5.r_1.fq.gz<br>HMGA1_H1299_DoxshHMGA1_2.s_4.r_1.fq.gz HMGA1_H1299_DoxshHMGA1_2.s_5.r_1.fq.gz<br>HMGA1_H1299_DoxshHMGA1_3.s_4.r_1.fq.gz HMGA1_H1299_DoxshHMGA1_3.s_5.r_1.fq.gz<br>Input_H1299_DoxshHMGA1_1.s_4.r_1.fq.gz Input_H1299_DoxshHMGA1_1.s_5.r_1.fq.gz<br>Input_H1299_DoxshHMGA1_2.s_4.r_1.fq.gz Input_H1299_DoxshHMGA1_2.s_5.r_1.fq.gz<br>Input_H1299_DoxshHMGA1_3.s_4.r_1.fq.gz Input_H1299_DoxshHMGA1_3.s_5.r_1.fq.gz<br>H1299-4C-HMGA1-1_1.fq.gz H1299-4C-HMGA1-1_2.fq.gz<br>H1299-4C-HMGA1-2_1.fq.gz H1299-4C-HMGA1-2_2.fq.gz<br>H1299-RT-HMGA1-1_1.fq.gz H1299-RT-HMGA1-1_2.fq.gz<br>H1299-RT-HMGA1-2_1.fq.gz H1299-RT-HMGA1-2_2.fq.gz<br>H1299-RT-input_1.fq.gz H1299-RT-input_2.fq.gz<br>HMGA1KO-4C-HMGA1-1_1.fq.gz HMGA1KO-4C-HMGA1-1_2.fq.gz<br>HMGA1KO-4C-HMGA1-2_1.fq.gz HMGA1KO-4C-HMGA1-2_2.fq.gz<br>HMGA1KO-RT-HMGA1-1_1.fq.gz HMGA1KO-RT-HMGA1-1_2.fq.gz<br>HMGA1KO-RT-HMGA1-2_1.fq.gz HMGA1KO-RT-HMGA1-2_2.fq.gz<br>HMGA1KO-RT-input-1_1.fq.gz HMGA1KO-RT-input-1_2.fq.gz<br>HMGA1KO-RT-input-2_1.fq.gz HMGA1KO-RT-input-2_2.fq.gz<br>Grow_HMGA1_1.bw<br>Grow_HMGA1_2.bw<br>Grow_HMGA1_1.bw<br>Grow_HMGA1_2.bw<br>RIS_HMGA1_1.bw<br>RIS_HMGA1_2.bw<br>RIS_HMGA1_1.bw<br>RIS_HMGA1_2.bw<br>HMGA1_H1299_1.bw<br>HMGA1_H1299_2.bw<br>HMGA1_H1299_3.bw<br>HMGA1_H1299_1.bw<br>HMGA1_H1299_2.bw<br>HMGA1_H1299_3.bw<br>HMGA1_H1299_DoxshHMGA1_1.bw<br>HMGA1_H1299_DoxshHMGA1_2.bw<br>HMGA1_H1299_DoxshHMGA1_3.bw<br>HMGA1_H1299_DoxshHMGA1_1.bw<br>HMGA1_H1299_DoxshHMGA1_2.bw<br>HMGA1_H1299_DoxshHMGA1_3.bw<br>H1299-4C-HMGA1-1.bw<br>H1299-4C-HMGA1-2.bw<br>H1299-RT-HMGA1-1.bw<br>H1299-RT-HMGA1-2.bw<br>H1299-RT-input.bw<br>HMGA1KO-4C-HMGA1-1.bw<br>HMGA1KO-4C-HMGA1-2.bw<br>HMGA1KO-RT-HMGA1-1.bw<br>HMGA1KO-RT-HMGA1-2.bw<br>HMGA1KO-RT-input-1.bw<br>HMGA1KO-RT-input-2.bw

Genome browser session
(e.g. UCSC)

Tracks with normalized ChIP-seq signal (THOR: library normalized and input subtracted) were submitted to GEO which can be downloaded and visualized with the genome browser of choice.

Methodology

Replicates

For each condition tested (Growing IMR90, RAS-induced senescence IMR90, H1299 with and without HMGA1 knock-down, H1299 with HMGA1 KO at different fixation conditions - 4C or room temperature) either two or three biological replicates were available and matching input files.

Sequencing depth

Single-end (50bp reads): (total reads / unique reads)
Grow_HMGA1_1 144602990 134822761
Grow_HMGA1_2 158640173 143312593
Grow_Input_1 70042429 65553776
Grow_Input_2 62090516 58036994
RIS_HMGA1_1 125748741 118215544
RIS_HMGA1_2 118552025 110599612
RIS_Input_1 64558856 60331464
RIS_Input_2 54731311 51040019
HMGA1_H1299_1 50281409 40670690
HMGA1_H1299_2 62124067 48795432
HMGA1_H1299_3 57163510 45125266
HMGA1_H1299_Dox_1 13338486 10707623
HMGA1_H1299_Dox_2 62835590 49820378
HMGA1_H1299_Dox_3 45753862 36768960
Input_H1299_1 59443346 44689949
Input_H1299_2 58852501 45070555
Input_H1299_3 67236806 50634056
Input_H1299_Dox_1 183406414 136413215
Input_H1299_Dox_2 71907291 53206808
Input_H1299_Dox_3 76864134 58056672

Paired-end (50bp reads): (total reads / unique reads)
H1299-4C-HMGA1-1 45865647 40783442
H1299-4C-HMGA1-2 54889065 48683370
H1299-RT-HMGA1-1 55663457 49427683
H1299-RT-HMGA1-2 50922538 44967073
H1299 Input 40204989 35208413
HMGA1KO-4C-HMGA1-1 45679387 39347302
HMGA1KO-4C-HMGA1-2 40975631 37052916
HMGA1KO-RT-HMGA1-1 60091232 51876560
HMGA1KO-RT-HMGA1-2 49572381 44741426
HMGA1KO-RT-input-1 51794652 45193519
HMGA1KO-RT-input-2 53802824 46902523

Antibodies

anti-HMGA1 (abcam ab192153, lot# GR3212620-2, 10 µg/50 M cells)

Peak calling parameters

Macs3 parameters: -t signal.bam -c input.bam -f BAM -g hs --call-summits
For the final peaks, we calculated the extension size with the ChIPQC R package and re-ran macs3 with --nomodel --extsize extension_size_calculated.

Data quality

We checked the initial libraries with FastQC for any sequencing artefacts and base sequence quality was very good with no adapter content detected so no trimming required. We next checked the percentage of uniquely aligned reads, the number of peaks called with macs3 and the number of peaks present in at least two replicates. We also calculated correlation coefficients between signal computed at different resolutions.

Peaks at FDR 0.05 and Fold change > 5 as required:
Grow_HMGA1_1 201348
Grow_HMGA1_2 257185
RIS_HMGA1_1 173060
RIS_HMGA1_2 186030
HMGA1_H1299_1 159363
HMGA1_H1299_2 165458
HMGA1_H1299_3 174992
HMGA1_H1299_Dox_1 47804
HMGA1_H1299_Dox_2 59748
HMGA1_H1299_Dox_3 57013
HMGA1_4C_H1299_1 356301
HMGA1_4C_H1299_2 341917
HMGA1_RT_H1299_1 312975
HMGA1_RT_H1299_2 280731
HMGA1KO_4C_H1299_1 0
HMGA1KO_4C_H1299_2 9987
HMGA1KO_RT_H1299_1 0

HMGA1KO_RT_H1299_2 8983

Software

Reads were aligned using Bowtie2 (v2.2.4) [Langmead et al. 2012 22388286] against the hg19 genome build. No trimming was required as checked with FastQC (v0.12) [Simon Andrews]. Duplicates were marked with samtools (v1.9) [Danecek2021 33590861] and blacklisted regions (hg19) were removed [Amemiya et al. 2019 31249361]. Samples were checked for quality control metrics and extension sizes were calculated for each sample with the R Bioconductor package ChIPQC (v.1.32.2)[Caroll2014 24782889]. Peaks were called with macs3 (v.3.0.0) initially with default parameters and then with the extension sizes determined. Peaks present in at least two of the replicates of a condition were combined into a consensus set for each condition. THOR (v.1.0.2) [Alhoff et al. 2016 27484474] was used to normalise and to perform differential binding pairwise between conditions. The THOR-normalised signal was used for visualisation with the IGV (v.2.16.2) genome browser [Robinson et al. 2011 21221095]. ChIP-seq heatmaps were plotted using deepTools (v.3.1.0) [Ramirez et al. 2016 27079975]. Peak annotation was performed with the R Bioconductor package annotatR (v.1.22.0) [Cavalcante et al. 2017 28369316].
